# A Virtual Reality Resident Training Curriculum on Behavioral Health Anticipatory Guidance: Development and Usability Study

**DOI:** 10.2196/29518

**Published:** 2021-06-29

**Authors:** Rachel Herbst, Tiffany Rybak, Andrea Meisman, Monica Whitehead, Brittany Rosen, Lori E Crosby, Melissa D Klein, Francis J Real

**Affiliations:** 1 Division of Behavioral Medicine and Clinical Psychology Cincinnati Children's Hospital Medical Center Cincinnati, OH United States; 2 Department of Pediatrics University of Cincinnati College of Medicine Cincinnati, OH United States; 3 Division of Adolescent and Transition Medicine Cincinnati Children’s Hospital Medical Center Cincinnati, OH United States; 4 Division of General and Community Pediatrics Cincinnati Children’s Hospital Medical Center Cincinnati, OH United States

**Keywords:** resident education, virtual reality, behavioral health promotion, COVID-19

## Abstract

**Background:**

Behavioral health disorders have steadily increased and been exacerbated by the COVID-19 pandemic. Though behavioral health disorders can be successfully mitigated with early implementation of evidence-based parent management strategies, education for pediatric residents on behavioral health anticipatory guidance has been limited to date, with training challenges compounded by the physical distancing requirements of the COVID-19 pandemic. Virtual reality (VR) simulations provide an opportunity to train residents on this complex competency by allowing deliberate practice of necessary skills while adhering to current social distancing guidelines.

**Objective:**

This study explored the usability of a VR-based behavioral health anticipatory guidance curriculum for pediatric residents.

**Methods:**

This mixed methods study included 14 postgraduate third-year pediatric residents who completed the behavioral health anticipatory guidance VR curriculum. Residents completed the MEC Spatial Presence Questionnaire to assess immersion in the virtual environment. Semistructured interviews were used to elucidate residents’ perspectives on the curriculum’s content and format. The interviews were analyzed using conventional content analysis.

**Results:**

Quantitatively, residents reported a high degree of immersion, spatial presence, and cognitive involvement. Most residents (11/14, 79%) agreed or strongly agreed that it seemed as though they took part in the action of the simulation. Qualitatively, two themes emerged from the data: (1) the curriculum expands behavioral health anticipatory guidance and motivational interviewing knowledge and skills and (2) VR technology is uniquely positioned to develop competence. These themes revealed that the curriculum expanded their current level of knowledge and skill, addressed training gaps, and was applicable to all residents. Additionally, residents experienced VR as immersive, feasible, realistic to the clinic setting, and a safe space to practice and learn new skills.

**Conclusions:**

Pilot data indicates that VR may be an effective tool to teach pediatric residents behavioral health anticipatory guidance, meeting a current gap in medical education training. This VR curriculum is particularly relevant in the context of the COVID-19 pandemic given the increased behavioral health concerns of families.

## Introduction

### Background

The behavioral health trajectory of children and youth in the United States is troubling [1,2], with 15% of children currently diagnosed with a behavioral health disorder that affects their mental and physical health during childhood and into adulthood [3,4]. The emergence of behavioral health concerns begins early in life, as do behavioral health promotion opportunities [5,6]. The development of behavioral health disorders may be mitigated with the implementation of evidence-based parenting strategies that address typical childhood behaviors. Although the COVID-19 pandemic has limited traditional sources of caregiver support, such as social networks, pediatric primary care remains a safe and trusted setting to address behavior concerns and decrease parental stress using evidence-based behavioral health anticipatory guidance [7]. Behavioral health anticipatory guidance refers to recommendations that a clinician shares with a caregiver to support optimal child development and health, such as introducing and modeling the concept of praise to reinforce behaviors. Behavioral health anticipatory guidance can serve as a powerful tool to mitigate negative outcomes when coupled with motivational interviewing skills, which enhance motivation for action [1,2]. Despite its potential for significant impact on childhood health, curricula that effectively train providers on administering behavioral health anticipatory guidance have been limited to date, providing an opportunity to use novel technology to address this educational gap.

Prior to the COVID-19 pandemic, behavioral concerns constituted more than 30% of pediatric primary care encounters [6,8,9]. The COVID-19 pandemic has exacerbated child behavioral health disorders due to increased family stress, isolation, and financial hardship [10]. Similarly, disruption of routines is expected to increase the prevalence and severity of behavioral concerns [7,11,12]. This context coupled with increased caregiver stress may result in caregivers not responding to behaviors in the most effective manner. These factors suggest that, now more than ever, behavioral concerns will present in the primary care setting. Thus, ample opportunities exist to engage in behavioral health promotion and prevention efforts.

Bright Futures is the American Academy of Pediatrics’ set of evidence-based anticipatory guidelines for pediatric primary care in health promotion in the context of family-centered care [13]. However, current pediatric residency training infrastructure regarding the development and evaluation of behavioral health anticipatory guidance skills remains insufficient to prepare pediatric residents to effectively and efficiently identify, promote, and manage common pediatric behavioral health concerns [14-16]. This training gap impedes resident competence in applying social-behavioral science to patient care while attending to contextual factors, counseling families, and effectively communicating with diverse patient populations [17-19], as recommended by the American Academy of Pediatrics and the Accreditation Council of Graduate Medical Education. The American Academy of Pediatrics encourages facilitating enhanced communication skills, promoting social-emotional development in training, and incorporating mental health specialists in teaching clinics to ensure learners engage in mental health care [18]. In fact, the American Board of Pediatrics emphasizes the importance of pediatric providers in preventing and managing behavioral health by inclusion in several entrustable professional activities (EPA; EPA-6 [medical home] and EPA-9 [mental/behavioral health problems]) [20]. Ideally, residents would demonstrate competence prior to graduation and board certification.

Despite these recommendations and research supporting the primary care provider’s role in behavioral health promotion, limited guidance exists regarding the development of behavioral health anticipatory guidance skills [8,9]. Effective evaluation of curricula aimed at developing these competencies is crucial to effective skill acquisition. Direct observation is a common strategy to assess residents’ skills and provide feedback; however, it can be challenging to predict when behavioral concerns might naturally arise during the course of a clinical encounter. Moreover, limitations on supervising clinicians’ time and productivity makes direct observation less feasible in real-world settings. In addition, the COVID-19 pandemic has further limited opportunities for direct observation due to social distancing guidelines and decreased patient encounters for routine preventative care [7]. Inadequate preparation in delivering behavioral health anticipatory guidance holds unique implications for medical trainees, as research indicates that deficient training negatively impacts future care provision [17,21].

Technology may offer opportunities to address current barriers to residents’ education on administration of behavioral health anticipatory guidance while adhering to public health measures during the COVID-19 pandemic. Virtual reality (VR), a computer-generated environment where users interact with graphical characters called avatars, has demonstrated success with clinician training on communication skills including delivering bad news, addressing vaccine hesitancy, and working within interprofessional teams [22-24]. Within VR, a facilitator designs different scenarios based on specific training needs to promote deliberate practice, a goal-oriented method for skill development. Deliberate practice, characterized by engaging in the task, receiving immediate feedback, then repeating the task while incorporating this feedback [25], has demonstrated effectiveness in pediatric education but has not been evaluated in behavioral health training [26,27].

Implementation and evaluation of resident training on behavioral health anticipatory guidance using VR is novel; thus, usability testing, defined as factors affecting participants’ experience in using the device for its intended purpose, is an important first step to understand its strengths and weaknesses and determine if the curriculum is achieving its aims [28-32]. Thus, we conducted a mixed methods study assessing the usability of our VR curriculum, Promoting Resilience and Emotional health through Virtual Education iN Training (PREVENT), which provides a virtual environment for deliberate practice of providing behavioral health anticipatory guidance, among a cohort of postgraduate third-year pediatric residents. Exploring residents’ perspectives on PREVENT is critical to assessing its potential to support skill acquisition and address the educational gap related to training on this important topic in childhood health.

### Study Goals

This mixed methods, single-site study explores the usability and utility of PREVENT—a novel behavioral health training curriculum that uses VR simulations—to address current educational gaps related to behavioral health anticipatory guidance skills. We hypothesized that residents would find the VR training curriculum useful, immersive, and applicable to clinical practice.

## Methods

### Study Setting and Participants

The study was conducted in an institution with one of the largest pediatric residency training programs in the country, which trains approximately 204 residents per year. The urban pediatric primary care clinic at which the usability testing occurred is the continuity clinic site for approximately 84 residents and serves as the medical home for 19,000 patients, with approximately 33,000 visits annually. The patients are predominantly African American (75%) and publicly insured (90%). Approximately 40% of graduating residents pursue a career in general pediatrics.

Pediatric postgraduate third-year residents were eligible to participate. Recruitment was limited to senior residents due to their familiarity with common behavioral health concerns in primary care and willingness to provide their perspective on PREVENT’s ability to meet behavioral health training gaps. Participants were informed that this was an educational study that was not tied to their evaluation process in the resident training program. The study was deemed exempt by the Cincinnati Children’s Hospital Medical Center’s institutional review board. Consent was obtained from each participant prior to participation.

### Resident Training Curriculum

PREVENT used VR simulations as the primary educational strategy to enhance learners’ competence in behavioral health anticipatory guidance and foundational motivational interviewing skills. Motivational interviewing skills were included in the curriculum as successful administration of behavioral health anticipatory guidance requires use of motivational interviewing to build rapport and enhance motivation for action. Preparation to participate in the VR simulations included the review of four 15-minute didactic presentations, which provided content on development, behavioral management, and motivational interviewing principles. For the VR simulations, participants counseled caregiver avatars regarding typical behavior concerns for a 3-year-old child. All scenarios featured a caregiver with concerns about tantrums causing stress and an expressed desire for behavior management strategies. Each scenario was associated with learning objectives related to specific behavioral health anticipatory guidance and motivational interviewing skills (Multimedia Appendix 1). A collaborative team of pediatricians, psychologists, experts in health professional education, and technologists developed the scenarios in an iterative fashion that employed a specific algorithm for each scenario to promote standardization of the experience between learners.

**Figure 1:**
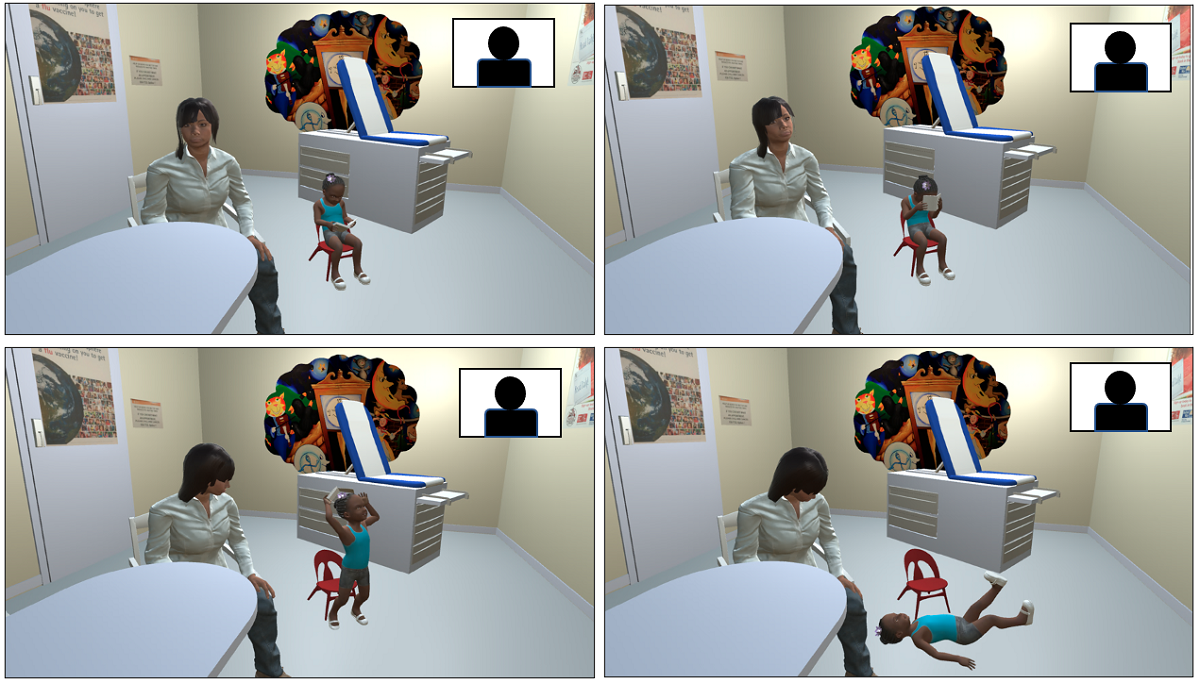
PREVENT included real-time interaction with an avatar family via the Zoom teleconferencing platform. Avatars were able to assume a number of different body positions including the ability for the child avatar to throw a tantrum during the clinical encounter. PREVENT: Promoting Resilience and Emotional health through Virtual Education iN Training.

The virtual visits occurred in an environment that replicated an actual examination room. The caregiver and child avatars were designed to reflect common caregiver and patient demographics and characteristics at our primary care center, including physical appearance (ie, race, gender), spoken language, and nonverbal cues. Avatars assumed a range of body positions to indicate different emotions, and audio was recorded and synchronized with the avatars’ facial expressions and mouth movements. Across the three scenarios, the resident participant counseled the avatar family on behavioral concerns and the avatar caregiver responded in real time in a realistic manner driven by a single facilitator (FJR). As the COVID-19 pandemic meant that residents could not participate in person via a VR headset, all sessions were completed on Zoom, a cloud-based videoconferencing service that allows screen sharing and secure recording. Participants accessed Zoom via an internet-capable device (eg, laptop, tablet, mobile phone) and were able to view the virtual environment and interact with the avatars (Figure 1). Following each scenario, the resident received feedback from the facilitator (FJR) about their demonstration of learning objectives. If residents did not meet the learning objectives for the case, they repeated the case to deliberately practice specific skills by incorporating the facilitator’s feedback [25]. The scenarios were scaffolded to increase in difficulty and complexity, building on the participant’s baseline skills over time. Details of the scenarios and corresponding learning objectives are described in Multimedia Appendix 1. The three scenarios and corresponding feedback were completed in approximately 30 minutes.

Participation in virtual patient simulators via a computer screen is consistent with the approach of many prior VR curricula targeting communication skills [23]. Our team previously demonstrated the impact of immersive 3D VR training on residents’ communication skills related to addressing influenza vaccine hesitancy; however, the benefits of 3D versus 2D virtual environments for medical communication training in this population require further investigation [22].

### Data Collection

Following the completion of the PREVENT curriculum, participants completed the MEC Spatial Presence Questionnaire to assess presence and immersion in the virtual environment [33]. This instrument uses a 5-point Likert scale ranging from strongly disagree (1) to strongly agree (5). The instrument has prior validity evidence and has previously been used to assess immersion in virtual medical curricula [34].

A semistructured interview guide was used to assess residents’ perceptions of the usability of PREVENT (Multimedia Appendix 2). Interview guide questions were adapted from usability testing literature [35-37]. The interview primarily queried participants about three topics: (1) residents’ preparation for, learning from, and recommended changes to the curriculum, (2) usability of Zoom (eg, ease of participation and use of technology), and (3) ability to be immersed in VR and any associated side effects of the technology. At the end of each interview, the researcher confirmed all relevant information had been included to ensure the quality and accuracy of the data. All interviews were recorded, transcribed, verified for accuracy, and entered into the qualitative analysis software ATLAS.ti (ATLAS.ti Scientific Software Development GmbH) [38].

### Statistical Analysis

#### Quantitative

We used descriptive and summary statistics for participant demographics and scores on the MEC Spatial Presence Questionnaire.

#### Qualitative

The principal investigator (RH), a psychology postdoctoral fellow (TR), and a research assistant (AM) analyzed the interview data using an inductive approach via conventional qualitative content analysis as outlined by Hsieh et al [39]. This methodological approach mirrors traditional thematic analysis as it focuses on identification of repeated patterns of meaning across a data set and differs from summative content analysis, which focuses primarily on counts and quantification of data [40].

All three individuals have had advanced training related to behavioral health and have prior experience conducting qualitative research. The researchers independently reviewed 20% of the interviews to obtain an overall framework of residents’ experiences with PREVENT. The researchers subsequently coded the key concepts in this subset of transcripts that aligned with the interview questions [39]. Once the data were independently coded from the subset, the researchers discussed preliminary findings, as the use of multiple analysts to contribute to the development of codes enhances the findings’ credibility [41]. Codes were added or revised to reflect emerging patterns in the data. After the development of consensus around these initial codes, the researchers independently coded the remaining transcripts. Coding led to the formation of categories and subsequently principal themes. Differences between researchers were resolved by discussing the underlying meanings of categories, revisiting the data, and reflecting on underlying elements to reach consensus. Saturation was reached when no new codes emerged. Coding checks (ie, ongoing comparisons of independent coding, discussions to rectify differences) with two independent coders resulted in eventual consistency of greater than 90%, indicating further review was not necessary [41].

To promote credibility, the researchers reflected on potential sources of bias. These included the researchers’ mental health training and emphasis on promoting behavioral health—which may have influenced the clarifying questions asked during the interviews and interpretation of participant responses—and the researchers’ role in developing and implementing PREVENT. The principal investigator completed an independent audit of 20% of transcripts to verify the credibility of the coding. Using two independent coders and credibility checks with participants were strategies employed to address and minimize potential bias and positionality while optimizing accurate representation of participants’ perceptions [41]. One resident participant reviewed the categories following data analysis to assess alignment with their personal experience of having completed the PREVENT curriculum (ie, member checking).

## Results

### Overview

A total of 36 categorical pediatric postgraduate third-year residents were eligible to participate and 14 (39%) enrolled in the study to complete PREVENT and provide data regarding its usability. Participants had a mean age of 29.4 years (Table 1). Of the 14 participants, 11 were female (79%), 11 were Caucasian (79%), and 10 were non-Hispanic (92%).

**Table 1:** Participant demographic data (N=14).

Characteristic		Participants
Age (years), mean (SD)		29.4 (2.6)
**Sex, n (%)**		
	Male	3 (21)
	Female	11 (79)
**Race, n (%)**		
	Caucasian	11 (79)
	Asian	1 (7)
	Other	1 (7)
	Prefer not to answer	1 (7)
**Ethnicity, n (%)**		
	Hispanic	1 (7)
	Non-Hispanic	13 (93)

### Quantitative

On the MEC Spatial Presence Questionnaire, 100% of residents (14/14) agreed or strongly agreed that they could devote their whole attention to the VR experience. There were 11 residents (79%) who agreed or strongly agreed that the VR experience captured their senses and that it seemed as though they actually took part in the action of the presentation. A total of 8 residents (57%) reported that it felt like they were actually in the environment of the presentation. There were 12 residents (86%) who agreed or strongly agreed that the VR presentation activated their thinking. In addition, 7 participants (50%) disagreed or strongly disagreed that they felt they could do things with the objects in the virtual presentation (Multimedia Appendix 3).

### Qualitative

In this study, two themes emerged from the data: (1) the PREVENT curriculum expands behavioral health anticipatory guidance and motivational interviewing knowledge and skills and (2) VR technology is uniquely positioned to develop competence. These themes encompassed residents’ perspectives on the purpose of PREVENT, the curriculum’s structure, and the use of the VR modality for instruction. Table 2 provides an overview of themes, categories, and codes, as well as supporting participant quotes.

**Table 2:** Qualitative results from resident interviews.

Theme, category, and code			Supporting participant quotes
**Theme 1. PREVENT^a^ expands behavioral health anticipatory guidance and motivational interviewing knowledge and skills**			
	**Category 1. Building on previous experiences and didactic knowledge**		
		Beyond medical school curriculum	“The general MI [motivational interviewing] concepts - we learned that in med school. But trying to incorporate in family interactions, that’s next level that I really liked. That was new for me.” “So all of the things that I feel really knowledgeable about with medication management and things are not always the most common things that parents are worried about or concerned about. So having a little bit extra after medical school to help give real advice could be helpful.”
		Preparedness for activity	“Pre-clinic [didactic] teaching definitely gets the baseline and I have it fresh in my mind. So, for the spanking example, I remember that lecture pre-clinic, and then I had a parent who spanked, so I could immediately use what was just learned.”
	**Category 2. PREVENT addressed training gaps**		
		Helpful and practical	“I was pleasantly surprised with what we were able to do. By the second visit, there seems to be about four to five sort of set responses... At the same time, I know that visits can go different ways, and so by the last visit when the kid was acting up, I was very pleasantly surprised by that, because I'm like, okay, let me reset here and figure out what I want to do next. And I think that's a very recurring theme in clinic, where you're like, okay, that was a curveball. For example, when you threw the corporal punishment on me, I wasn't expecting to talk about this. Let me go back and bring out what I like to do in these cases.”
		Length of experience	“It was very engaging and active participation on my part. I was able to address multiple skills in the time. So, I thought it was just right.”
		Depth of content and skills	“I liked when you were coaching us, like what to talk about and just kind of reviewing briefly different methods of trying to like redirect, trying to ignore, things like that. It was giving a refresher as well as giving examples. It was like nice and short and sweet, not too long, but high yield.”
		Scaffolding and repetition	“It was super helpful. Going from visit to visit made this scenario [say] can you add this into the mix? It's a little challenging to integrate things, and it's also an immediate technique to improve yourself. Because hopefully someone is coming in and asking open ended questions, asking permission [then] let's work on redirection. Let's work on these other techniques. Integrating that as you're going forward can only be helpful, because oftentimes it's easy [for the preceptor] to say ‘next time, try not to do this, this, and this.’ Then you just don't practice it.”
	**Category 3. Unique benefits of PREVENT**		
		Safe	“I think it creates this safe space where, even if I know you’re listening, I’m just focused on ‘this is the roleplay I’m doing. I don’t know this cartoon person. They’re not judging me.’” “I liked that it was not real patients. Sometimes I think experimenting on real people feels wrong or just a little bit icky feeling, so it was nice to be able to practice and make mistakes when the stakes were lower.”
		Recommend and applicability	“I recommend [PREVENT] because this was a positive experience. It was a minimal time commitment, it doesn’t have the social pressure of real people, and I was able to gain new skills in a short amount of time.” “It's good practice and you get feedback that could change your practice. If you were going into private practice, you'd probably be having a lot more of these conversations than I've had as a resident. And I would not feel prepared to be having those conversations.”
		Applicability to all pediatric residents	“Even if they’re not going to be primary care physicians, they should all have these skills and they can utilize them in all the different [pediatric] specialties.”
**Theme 2. Virtual reality technology is uniquely positioned to develop competence**			
	**Category 4. Technology was feasible**		
		Ease of use	“I love how you've transitioned over for our VR. It gives us a lot of flexibility in what we're able to do and especially because you have us [the residents] really engaged in the curriculum/software, you know.” “I actually haven’t used Zoom before this, and so I just like downloaded it, and it was super easy.”
		Facilitation of activity	“I like to see a diagram of things, so I thought that visualizing [resident feedback] in a slide was really nice.”
		Transportability	“It [was transportable] specifically for me on maternity leave, but more for the whole coronavirus, this works well. It could be good things for people to use to continue [to learn].” “Really easy, and it took away barriers. [For people] wanting to do it, it was more flexible. I’m on my ER rotation now, so I have time in between shifts, so it was an easy enough way to participate.”
		Side effects	“No, no side effects. It all worked out great.”
		Zoom compared to virtual reality	“Yeah, and [VR] was neat, because you could control looking around the room, and maybe especially in this scenario, where the kid is like having those behaviors... But I still felt it was effective on Zoom.”
	**Category 5. Realism of virtual reality similar to clinic and aided learning**		
		Immersive environment	“It's immersive enough that you're like, ‘all right, I'm taking this seriously.’ I'm in this visit. This is a real set of responses that I might get and let me see how I might address that. That's why I seriously appreciate this curriculum.” “I actually really liked that it mimicked PPC [clinic], because it just felt more comfortable, even the office looking the same... It just made it feel less like intimidating than going into some random like standardized room so I liked that it mimicked what we do every week at PPC [clinic].”
		Interactive and engaging	“The interactive nature of it [is helpful], so it’s not just reading off a script. They’re actually giving you different responses depending on what you ask. So, a little bit more flexible than just a set script.”
		Nonverbals/body language	“I think I didn’t pick up on like a huge amount from the parent. I think there was some that just showed that she was kind of interacting with me, but I didn’t get a huge sense of like ‘Oh, she’s leaning in now, so she’s more engaged like, oh, she’s pulling away, so she’s less.’ But the tantrum that the child had in clinic was well done.”

^a^PREVENT: Promoting Resilience and Emotional health through Virtual Education iN Training.

#### Theme 1: PREVENT Expands Behavioral Health Anticipatory Guidance and Motivational Interviewing Knowledge and Skills

##### Overview

Participants articulated how the curriculum built upon their previous experiences in medical school and residency to develop competence in managing common behavioral presenting concerns. This theme captured how the unique structure of PREVENT, with the inclusion of both behavioral health anticipatory guidance and motivational interviewing skills in a singular curriculum, the use of deliberate practice, and safe and accessible VR technology, was crucial to PREVENT’s usability. Residents also indicated awareness of their prior limited skills in behavioral health anticipatory guidance and motivational interviewing, which made them doubt their competence to support families’ behavioral health concerns prior to participation in PREVENT. Theme 1 encompassed three categories.

##### Category 1: Building on Previous Experiences and Didactic Knowledge

Participants described how PREVENT expanded their motivational interviewing and behavioral health anticipatory guidance knowledge and skills beyond previous educational activities. Specifically, they described limited medical school training, while noting these are the topics that “we have the least experience in coming out of medical school, but it's [behavioral concerns] a super frequent problem with patients in the primary care setting.” In addition, participants indicated that the didactic teaching on motivational interviewing and behavioral health anticipatory guidance provided sufficient foundational knowledge to prepare them to participate meaningfully in the VR scenarios.

##### Category 2: PREVENT Addressed Training Gaps

Participants shared the unique benefits of VR as an educational strategy. Anchored in the recognition that the motivational interviewing and behavioral health anticipatory guidance skills used in VR are directly translatable to actual clinical experience, participants described how PREVENT met learning goals and scaffolded skill development. One participant shared “I think it was a great practice strategy for refreshing me on what I already know but then getting more of those [motivational interviewing and behavioral health anticipatory guidance] skills.” Specifically, participants noted that the length of the experience, depth of content and skills, and structured repetition over increasingly challenging scenarios provided opportunities for deliberate practice that enhanced their motivational interviewing and behavioral health anticipatory guidance competences.

##### Category 3: Unique Benefits of PREVENT

Participants indicated that the training met their educational needs and VR provided an opportunity to learn in a safe space: “I liked that it was not real patients…sometimes I think experimenting on real people feels wrong or just a little bit icky feeling, so it was nice to be able to practice and make mistakes when the stakes were lower.” In addition, participants highly recommended the training for other residents, describing PREVENT’s applicability to all pediatric residents. “I wish I had this during intern year ... I [am] actually trying to put this into practice and I could have gotten further [with my skill development] ... I could have learned throughout my residency by really advancing my motivational skills.”

#### Theme 2: VR Technology is Uniquely Positioned to Develop Competencies

##### Overview

This theme captured how PREVENT’s VR technology increased engagement through immersion and created an ideal learning environment that was easy to access and use, uniquely facilitating residents’ development of skills and competencies. Theme 2 encompassed categories 4 and 5.

##### Category 4: Technology Was Feasible

Participants outlined the benefits of VR. Specifically, they noted that VR is easy to use, comfortable to engage in across various settings (eg, at home, in a clinic room), and the behind-the-scenes facilitation was minimally apparent. All participants denied VR side effects. Finally, they reported that the VR experience via Zoom was sufficiently immersive and engaging. Those with previous VR experience perceived this VR training via Zoom as equally effective to VR training using a 3D-mounted headset.

##### Category 5: Realism of VR Similar to Clinic and Aided in Learning

Participants commented on the unique aspects of VR as an educational modality. Specifically, participants described how the immersive environment of PREVENT made interactions feel realistic. They indicated that this experience was augmented by the interactive nature of the avatars, such as avatar responses to participants’ language. Finally, some participants indicated that the nonverbal communication of the avatars felt realistic, while others reported that the nonverbal communication was subtle and that they desired more pronounced body language.

## Discussion

### Principal Results

Results from this usability study demonstrated PREVENT’s applicability to common presenting concerns and ease of use among pediatric residents. Quantitatively, resident participants in PREVENT reported a high level of immersion, spatial presence, and cognitive involvement. Qualitative data indicated that residents perceived PREVENT as effective in expanding their behavioral health anticipatory guidance and motivational interviewing knowledge and skills by providing the appropriate breadth and depth of content and effectively employing deliberate practice to promote skill development. Residents indicated that the curriculum was applicable to real-world practice and balanced the depth of skill development with a feasible length of training. The use of Zoom, which arose because of COVID-19 restrictions, was well received and reported as sufficiently immersive for the specific learning objectives of PREVENT.

Participants underscored PREVENT’s applicability to all levels of pediatric residents, regardless of training year or pediatric subspecialty, and reported that PREVENT expanded their competence to meet the behavioral health needs of families with young children. This is particularly crucial with recent increased family stress and the higher prevalence of behavioral health concerns secondary to COVID-19 [7]. This curriculum could be applicable and generalizable to other programs. The VR modality provided portability and transferability that circumvented the barriers associated with traditional face-to-face training in the context of physical distancing due to the pandemic. Due to its convenience and acceptability, VR programs may continue to be applicable to resident training after physical distancing restrictions are lifted. This study provided preliminary support for the effectiveness of transitioning VR from a 3D platform (VR headset) to a 2D platform (Zoom) for communication training given the reported sufficiency of attention allocation and spatial presence among participants.

Tantrums are a common presenting concern, but few opportunities exist to scaffold skills needed to coach a caregiver through managing a tantrum. Through PREVENT’s novel use of VR, we provided residents with an immersive and realistic educational opportunity to develop motivational interviewing and behavioral health anticipatory guidance skills, which are important and commonly used skills, but ones with limited prior opportunities to practice. The VR environment also enables the integration of motivational interviewing and behavioral health anticipatory guidance skills, which are required concurrently in actual clinical practice. The structure of increasingly complex scenarios with feedback between scenarios, a key element of deliberate practice, allowed for scaffolding of learning. Although deliberate practice may require more infrastructure and resources than other educational modalities attempting to promote behavior change (eg, webinars) [42,43], this approach (deliberate practice) appears to be a critical component of PREVENT’s perceived effectiveness.

The safe learning environment of PREVENT was an unanticipated study finding. Residents described their sense of incompetence and imposter syndrome prior to PREVENT. They described the expectation to use motivational interviewing and behavioral health anticipatory guidance skills, which are challenging skills to implement, as anxiety-provoking when limited opportunities for skill development exist. PREVENT offers a strategy to decrease resident stress through scaffolded experiences and a framework to use for collecting history and implementing interventions. The VR environment can help shape resident skills that resemble real-life encounters in a safe environment without patients, thus decreasing anxiety during practice and potentially decreasing stress when experienced in an actual clinical setting.

### Limitations and Strengths

This article has several limitations. First, as a single-site study, our results may lack transferability to other settings with different types of learners and resources. However, since we reached saturation with our qualitative results, we believe these may be transferable. Second, we only investigated the use of VR on behavioral health anticipatory guidance so our results may not be generalizable to other behavioral or mental health conditions. Third, as a pilot study, we investigated the usability of VR as an educational tool for behavioral health anticipatory guidance and motivational interviewing training but did not collect data on residents’ actual performance with patients. Finally, a social desirability bias may have been present during qualitative interviews, despite attempts to minimize this bias by emphasizing that honest responses would promote residents’ ability to help cocreate curriculum adaptations. Additional research is needed to study PREVENT’s effectiveness and impact on resident competence.

Due to these limitations, our next steps include performing a randomized controlled trial of our intervention to provide efficacy data. Longitudinal data and patient-reported outcomes will generate crucial learnings about the short- and long-term impact on skill development and behavioral health promotion. Future multisite trials will provide data about the generalizability and transferability of the curriculum to other training sites. The development of artificial intelligence is an important future step as it will eliminate the need for a human facilitator in the VR environment, allowing scalability and potentially decreasing cost. Finally, future research could explore testing PREVENT with scenarios focused on other behavioral health presenting concerns (eg, picky eating, sleep).

Despite these limitations, our study has several strengths. First, we used semistructured interviews, which allowed us to obtain both a breadth and depth of information about the content of the curriculum and the process of delivering PREVENT. We believe the interview guide explored important aspects of both the curriculum and the VR experience itself. Second, we were able to establish both credibility and trustworthiness in robust ways while reducing bias. Third, we selected an appropriate expert population for usability testing. Finally, we believe our study is particularly pertinent to the COVID-19 pandemic, given that our methods were executed virtually.

### Comparison With Prior Work

The curriculum meets training gaps identified by the pediatric residency training leadership, aligning with calls for action from American Academy of Pediatrics and American Board of Pediatrics for common factors approaches to emerging behavioral health concerns [15,17,18,20]. PREVENT builds competence in teaching parental behavioral health management skills that have been exclusively taught through parenting programs [44] and are vital to meeting family needs in the context of primary care. PREVENT also aligns with the calls for increased prevention and positive parenting in primary care through the development and implementation of training initiatives [5].

Finally, the use of VR in medical education to increase health care providers’ communication skills and subsequently change practice behaviors and impact health outcomes has been documented in vaccine uptake, although it has not been applied to behavioral health anticipatory guidance until now [22]. A recent systematic review of virtual patient simulators reported that effective curricula emphasized scaffolding and human feedback, positive features of PREVENT identified by our participants [23]. This work also underscores the importance of usability testing of VR applications prior to real-world deployment to establish quality and safety measures of the approach [31]. Our mixed methods design to usability testing may provide a practical roadmap for others.

### Conclusions

PREVENT provides promising usability data that VR may be an effective tool to train pediatric residents on behavioral health anticipatory guidance and motivational interviewing skills. The curriculum aligns with calls to action in the medical education training community. Finally, the curriculum’s focus is of increased importance in the context of COVID-19, as traditional face-to-face training opportunities are limited, while family behavioral health needs are increased.

## Appendix

Multimedia Appendix 1 Virtual reality scenarios were scaffolded with increasing complexity and difficulty over time to allow demonstration of advanced skills.

Multimedia Appendix 2 Usability postcurriculum questions.

Multimedia Appendix 3 The MEC-Spatial Presence Questionnaire, an instrument for assessing immersion in a virtual environment, utilizes a 5-point Likert scale from strongly disagree (1) to strongly agree (5). Individual item and subscale scores for resident participants in PREVENT indicated a high level of attention allocation, spatial presence, and cognitive involvement.

## Abbreviations

EPA: entrustable professional activities
PREVENT: Promoting Resilience and Emotional health through Virtual Education iN Training
VR: virtual reality
