# Supplementary material for: A Virtual Reality Resident Training Curriculum on Behavioral Health Anticipatory Guidance: Development and Usability Study
Source: JMIR Pediatr Parent. 2021 Jun 29;4(2):e29518. doi: 10.2196/29518 (PMC8244725; doi:10.2196/29518)
Supplement: Multimedia Appendix 1 [file pediatrics_v4i2e29518_app1.doc]

**Multimedia Appendix 1.** Virtual reality scenarios were scaffolded with increasing complexity and difficulty over time to allow demonstration of advanced skills.

| **Table 1: PREVENT Virtual Reality Simulations** | | |
| --- | --- | --- |
| **Setting**: Cincinnati Children’s Hospital Medical Center Pediatric Primary Care Clinic | | |
| **Scenario 1** | **Scenario 2** | **Scenario 3** |
| **Narrative**: 3 yo female patient presents for behavior concerns  **Characters**: parent and female child avatar  **Behavior Concerns:** tantrums  **Previous family strategies attempted**: “time out” done incorrectly  **Environmental Factors**: none  **Behavioral Health Anticipatory Guidance (BHAG) Content Goals:** Antecedent-Behavior-Consequences (ABCs) and positive parenting with labeled praise and active ignoring | **Narrative**: 3 yo female patient presents for behavior concerns  **Characters**: parent and female child avatar  **Behavior Concerns:** tantrums and mild aggression  **Previous family strategies attempted**: “time out” done correctly, spanking  **Environmental Factors**: lives in apartment, neighbors have been complaining about noise  **BHAG Content Goals:** ABCs, labeled praise and active ignoring, and redirection | **Narrative**: 3 yo female patient presents for behavior concerns  **Characters**: parent and female child avatar  **Behavior Concerns:** tantrums, mild aggression (hitting) and child avatar has tantrum during clinical visit  **Previous family strategies attempted**: “time out” done correctly, intermittent ignoring  **Environmental Factors**: lives in apartment, neighbors have been complaining about noise, and maternal depression  **BHAG Content Goals:** ABCs, labeled praise and active ignoring, redirection and coaches parent through tantrum that occurs during clinical visit |
| **Key Motivational Interviewing (MI) Skills:** open-ended questions, reflection, asking permission and giving information; avoid advising without permission, confronting, or directing | | |
