# Supplementary material for: A Virtual Reality Resident Training Curriculum on Behavioral Health Anticipatory Guidance: Development and Usability Study
Source: JMIR Pediatr Parent. 2021 Jun 29;4(2):e29518. doi: 10.2196/29518 (PMC8244725; doi:10.2196/29518)
Supplement: Multimedia Appendix 2 [file pediatrics_v4i2e29518_app2.doc]

**Multimedia Appendix 2**. Usability Post-Curriculum Questions

| **PREVENT Curriculum** |
| --- |
| *Interviewer*: We have now completed the PREVENT curriculum. I will now ask you questions regarding your experience with the curriculum.   1. What are your overall impressions of the PREVENT curriculum? 2. What three things did you like most? 3. What three things did you like least?    1. If you could make one significant change to this intervention, what change would you make? 4. Did the preclinic teachings prepare you for the VR simulation?    1. If not, what components were missing?    2. Did this complement other types of instruction that you have received on this topic (For example: talking with psychologists about cases)?    3. If so, how? 5. Was the feedback administered between sessions helpful?    1. Too much or too little?    2. Did you feel adequately prepared to participate in the next scenario?    3. Were expectations clear? 6. Did you think the duration of the VR experience was appropriate?    1. Too long? What should be removed?    2. Too short? What should be added? 7. Would you participate in a virtual reality intervention like this (PREVENT) in the future?    1. Please share more? 8. Would you recommend PREVENT to a colleague?    1. Why or why not? 9. How applicable are these behavioral health **prevention** skills to your work in continuity clinic? |
| **Usability of** Zoom^TM^ **:** |
| *Interviewer*: Thank you for your responses regarding your experience with the curriculum. I will now ask you questions regarding your experience with using the Zoom^TM^ platform to participate:  1. How easy was it to participate in PREVENT over Zoom^TM^ ?  2. Any challenges with using the Zoom^TM^ technology?  a) Trouble with the connection? Trouble with the view? The audio? The avatars’ movements or dialogue?  3. Where did you participate? How did it feel participating in that location?  4. Do you see advantages or disadvantages to conducting this education over Zoom^TM^ ? |
| **Immersion & Side Effects:** |
| 1. How did participating in the VR simulations make you feel?    1. Did you feel immersed?    2. Did it feel realistic?    3. Did you experience any side effects? (PROBES: distracted, anxious, at ease, motion sickness, etc.) |
