# Supplementary material for: A Virtual Reality Resident Training Curriculum on Behavioral Health Anticipatory Guidance: Development and Usability Study
Source: JMIR Pediatr Parent. 2021 Jun 29;4(2):e29518. doi: 10.2196/29518 (PMC8244725; doi:10.2196/29518)
Supplement: Multimedia Appendix 3 [file pediatrics_v4i2e29518_app3.doc]

**Multimedia Appendix 3.** The MEC-Spatial Presence Questionnaire, an instrument for assessing immersion in a virtual environment, utilizes a 5-point Likert scale from strongly disagree (1) to strongly agree (5). Individual item and subscale scores for resident participants in PREVENT indicated a high level of attention allocation, spatial presence, and cognitive involvement.

| MEC-Spatial Presence Questionnaire Item | | Mean (SD) |
| --- | --- | --- |
| **Attention Allocation** | |  |
|  | I devoted my whole attention to the virtual reality experience. | 4.50 (0.52) |
|  | I concentrated on the virtual reality experience. | 4.57 (0.65) |
|  | The virtual reality experience captured my senses. | 3.93 (0.62) |
|  | I dedicated myself completely to the virtual reality experience. | 4.21 (0.89) |
| **Spatial Situation Model** | |  |
|  | I was able to imagine the arrangement of the space presented in the virtual reality experience. | 4.50 (0.65) |
|  | I had a precise idea of the spatial surroundings presented in the virtual reality experience. | 3.86 (1.10) |
|  | I was able to make a good estimate of the size of the presented space. | 4.29 (0.73) |
|  | Even now, I still have a concrete mental image of the spatial environment. | 4.36 (0.74) |
| **Spatial Presence** | |  |
|  | I felt like I was actually there in the environment of the presentation. | 3.29 (1.14) |
|  | It was as though my true location had shifted into the environment in the presentation. | 3.29 (1.07) |
|  | I felt as though I was physically present in the environment of the presentation. | 2.93 (1.21) |
|  | It seemed as though I actually took part in the action of the presentation. | 3.57 (1.28) |
| **Spatial Presence: Possible Actions** | |  |
|  | I had the impression that I could be active in the environment of the presentation. | 3.29 (1.20) |
|  | I felt like I could move around among the objects in the presentation. | 2.57 (1.09) |
|  | The objects in the presentation gave me the feeling that I could do things with them. | 2.64 (1.34) |
|  | It seemed to me that I could do whatever I wanted in the environment of the presentation. | 2.50 (1.34) |
| **Higher Cognitive Involvement** | |  |
|  | I thought most about things having to do with the virtual reality experience. | 3.64 (0.93) |
|  | I thoroughly considered what the things in the presentation had to do with one another. | 3.86 (0.95) |
|  | The virtual reality presentation activated my thinking. | 4.14 (0.86) |
|  | I thought about whether the virtual reality experience could be of use to me. | 4.00 (1.04) |
| **Suspension of Disbelief** | |  |
|  | I concentrated on whether there were any inconsistencies in the virtual reality experience.^a^ | 3.43 (0.94) |
|  | I didn't really pay attention to the existence of errors or inconsistencies in the virtual reality experience. | 3.14 (0.95) |
|  | I took a critical viewpoint of the virtual reality experience.^a^ | 2.86 (0.95) |
|  | It was not important for me whether the virtual reality experience contained errors or contraindications. | 3.43 (0.85) |
| **Domain Specific Interest** | |  |
|  | I am generally interested in the topic of virtual reality. | 3.71 (0.91) |
|  | I have felt a strong affinity to the theme of virtual reality for a long time. | 2.64 (1.28) |
|  | There was already a fondness in me for the topic of virtual reality before I was exposed to it. | 3.00 (1.11) |
|  | I just love to think about the topic of virtual reality. | 2.50 (0.94) |
| **Visual Spatial Imagery** | |  |
|  | When someone shows me a blueprint, I am able to imagine the space easily. | 3.14 (1.03) |
|  | It's easy for me to negotiate a space in my mind without actually being there. | 3.36 (1.01) |
|  | When I read a text, I can usually easily imagine the arrangement of the objects described. | 3.57 (0.94) |
|  | When someone describes a space to me, it's usually very easy for me to imagine it clearly. | 3.43 (1.02) |
| **Overall MEC-SQ Subscales** | |  |
|  | Attention Allocation | 4.30 (0.71) |
|  | Spatial Situation Model | 4.25 (0.84) |
|  | Spatial Presence | 3.27 (1.17) |
|  | Spatial Presence: Possible Actions | 2.75 (1.25) |
|  | Higher Cognitive Involvement | 3.91 (0.94) |
|  | Suspension of Disbelief | 3.21 (0.93) |
|  | Domain Specific Interest | 2.96 (1.14) |
|  | Visual Spatial Imagery | 3.38 (0.97) |

^a^Reverse score based on MEC-Spatial Presence Questionnaire manual
